# RR6预后模型在中国芦可替尼治疗骨髓纤维化患者中的外部验证及增量价值评估：以总生存为结局

**DOI:** 10.3760/cma.j.cn121090-20250418-00188

**Published:** 2026-01

**Authors:** 宁宁 柳, 冰 李, 铁军 秦, 士强 曲, 丽娟 潘, 蒙 焦, 清妍 高, 金影 赵, 志坚 肖, 泽锋 徐

**Affiliations:** 1 中国医学科学院血液病医院（中国医学科学院血液学研究所），血液与健康全国重点实验室，国家血液系统疾病临床医学研究中心，细胞生态海河实验室，天津 300020 State Key Laboratory of Experimental Hematology, National Clinical Research Center for Blood Diseases, Haihe Laboratory of Cell Ecosystem, Institute of Hematology & Blood Diseases Hospital, Chinese Academy of Medical Sciences &Peking Union Medical College, Tianjin 300020, China; 2 天津医学健康研究院，天津 301600 Tianjin Institutes of Health Science, Tianjin 301600, China

**Keywords:** 骨髓纤维化, 芦可替尼, RR6预后模型, 基因突变, Myelofibrosis, Ruxolitinib, RR6 prognostic model, Gene mutation

## Abstract

**目的:**

在中国骨髓增殖性肿瘤（MPN）相关骨髓纤维化（MF）患者队列中，对芦可替尼治疗6个月后反应（RR6）预后模型进行外部验证，并探讨整合高风险分子突变（HMR^mt^）及RAS信号通路基因突变（RASp^mt^）后的增量预后价值。

**方法:**

回顾性纳入2016年5月至2024年2月期间在中国医学科学院血液病医院接受芦可替尼治疗的153例MPN相关MF患者，应用RR6模型对患者进行预后分组及总生存（OS）分析，绘制校准曲线评价模型的校准度。采用一致性指数（C-index）、时间依赖性曲线下面积（AUC）、净重新分类改善指数（NRI）与临床决策曲线（DCA），全面评估并比较RR6及其整合模型的预测效能与临床效用。

**结果:**

纳入153例MPN相关MF患者，其中原发性骨髓纤维化（PMF）114例（74.5％）、真性红细胞增多症后骨髓纤维化（post-PV MF）17例（11.1％）、原发性血小板增多症后骨髓纤维化（post-ET MF）22例（14.4％）。中位随访44.6（*IQR*：27.3～64.5）个月，截至末次随访，106例（69.3％）存活，94例（61.4％）仍在接受芦可替尼治疗。RR6模型将患者划分为低危组（28例，18.3％）、中危组（92例，60.1％）及高危组（33例，21.6％），各组中位OS期分别为未达到、未达到和32（95％ *CI*：24.0～39.5）个月（*P*＝0.012）。校准曲线显示RR6模型在前3年校准度良好。在102例有二代测序数据的患者中，RR6+HMR^mt^+RASp^mt^整合模型展现出最优的预测性能，C-index（0.735）与AUC均最高，NRI证实该模型在1、2年能显著改善风险重分类（*P*＝0.016、*P*<0.001），且DCA显示其具有显著的临床净获益。

**结论:**

本研究证实RR6模型在中国MF患者中具有良好的预后预测能力，可用于早期识别高危患者。联合HMR^mt^与RASp^mt^或能进一步提升模型的预测效能。

## Abstract (en)

**Objective:**

To validate the Response to Ruxolitinib After 6 Months（RR6）prognostic model in Chinese patients with myelofibrosis（MF）treated with ruxolitinib and assess its incremental prognostic value when integrated with high molecular risk mutations（HMR^mt^）and/or RAS pathway mutations（RASp^mt^）.

**Methods:**

Altogether, 153 patients with myeloproliferative neoplasm-associated MF treated with ruxolitinib at our hospital from May 2016 to February 2024 were retrospectively enrolled. The RR6 prognostic model was applied for prognostic stratification and OS analysis, and calibration curves were plotted to evaluate the model's calibration. The predictive efficacy and clinical benefit of the RR6 model and its integrated models were comprehensively assessed and compared using the concordance index（C-index）, time-dependent area under the curve（AUC）, net reclassification improvement（NRI）, and decision curve analysis（DCA）.

**Results:**

Altogether, 153 patients were diagnosed with primary myelofibrosis（*n*＝114）, post-polycythemia vera（post-PV）MF（*n*＝17）, and post-essential thrombocythemia（post-ET）MF（*n*＝22）. Patients had a median follow-up time from the initiation of ruxolitinib treatment of 44.6（*IQR*: 27.3–64.5）months; 106（69.3％）patients survived, 47（30.7％）died, and 94（61.4％）were still receiving ruxolitinib as of the last follow-up. According to the RR6 model, 28（18.3％）, 92（60.1％）, and 33（21.6％）patients were classified as having low, intermediate, and high risks, respectively, with the estimated median overall survival（mOS）not reached for both the low- and intermediate-risk groups and 32（95％ *CI:* 24.0–39.5）months for the high-risk group（*P*＝0.012）. The calibration curves indicated that the RR6 model demonstrated good calibration performance at 1, 2, and 3 years. Among 102 patients with next-generation sequencing data, the integrated RR6+HMR^mt^+RASp^mt^ model demonstrated the best predictive efficacy, with the highest C-index（0.735）and AUC value. The NRI also confirmed that this model significantly improved risk reclassification at 1 and 2 years（*P*＝0.016, *P*<0.001）. Moreover, DCA showed significant net clinical benefit.

**Conclusion:**

The present study confirms that the RR6 prognostic model has favorable prognostic predictive ability in Chinese patients with MF and can be utilized for the early identification of high-risk patients. The integration of HMR^mt^ and RASp^mt^ may enhance the predictive power of the RR6 model.

Ph^-^骨髓增殖性肿瘤（Myeloproliferative neoplasms, MPN）相关骨髓纤维化（Myelofibrosis, MF）包括原发性骨髓纤维化（Primary myelofibrosis, PMF）、真性红细胞增多症后骨髓纤维化（Post-polycythemia vera myelofibrosis, post-PV MF）和原发性血小板增多症后骨髓纤维化（Post-essential thrombocythemia myelofibrosis, post-ET MF），为一组异质性疾病，患者可表现为进行性脾脏肿大和不同程度的血细胞减少[1]，芦可替尼治疗可缩小脾脏体积、改善体质性症状、延长生存[2]–[3]。新近Maffioli等[4]基于意大利人群数据，针对接受芦可替尼治疗的MPN相关MF患者提出了一个芦可替尼治疗6个月后反应（Response to ruxolitinib after 6 months, RR6）的预后模型，用于评估患者的生存预后，并早期识别可能因疗效不佳而需要及时调整治疗方案的高危患者。Coltro等[5]提出RR6与高风险分子突变（High molecular risk mutations, HMR^mt^）和RAS信号通路基因突变（RAS pathway mutations, RASp^mt^）整合后的预后预测能力得以进一步提高。然而，RR6及其整合模型在中国MPN相关MF患者群体中的适用性与预测效能如何，目前尚缺乏相关研究证据。本研究我们回顾性分析了153例接受芦可替尼治疗的MPN相关MF患者，旨在验证RR6模型在我国患者中的适用性，并评估其与HMR^mt^和（或）RASp^mt^整合后的预后预测能力。

## 研究方法

一、研究设计

本研究是一项用于外部验证的回顾性观察性研究，遵循针对个体预后或诊断多因素预测模型（Transparent Reporting of a Multivariable Prediction Model for Individual Prognosis or Diagnosis, TRIPOD）报告规范[6]。截至2024年2月，共检出345例在中国医学科学院血液病医院MDS和MPN中心连续收治并接受芦可替尼治疗的MPN相关MF患者，其中有258例PMF、36例post-PV MF和51例post-ET MF。根据入选标准（接受芦可替尼治疗时间不少于6个月、血小板计数>50×10^9^/L、关键指标完整、有随访记录），最终纳入共153例患者（图1），以此队列对RR6预后模型进行外部验证。RR6预后模型被作为一个完整的预测工具进行验证，我们未对其系数或变量进行重新估计或调整，具体评分标准见文献[4]，0分为低危组，1～2分为中危组，≥2.5分为高危组。

**图1:**
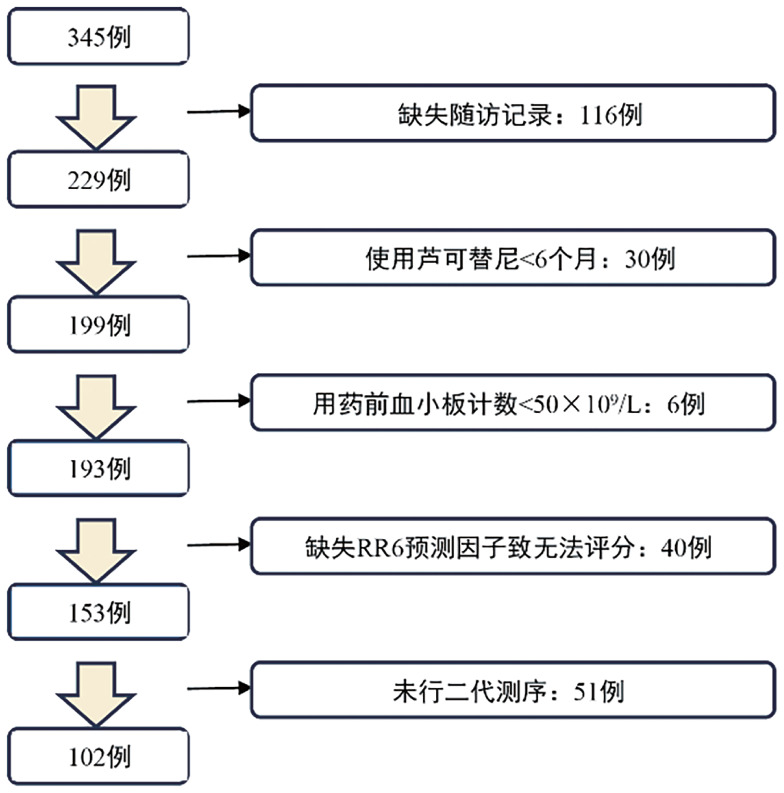
本验证队列患者入选流程图 **注** RR6：芦可替尼治疗6个月后反应

记录153例患者基线、第3个月和第6个月的红细胞输注情况、芦可替尼用量以及脾脏肋缘下长度。记录102例有二代测序（NGS）结果患者的HMR^mt^（包括ASXL1、EZH2、IDH1、IDH2、SRSF2或U2AF1）和RASp^mt^（包括NRAS、KRAS或CBL）情况。基因突变检测方法参见文献[7]。

所有患者均随访至2024年11月14日。随访资料来源于患者的门诊、住院病历资料以及电话随访记录。随访时间为自芦可替尼治疗起始至随访截止或死亡时间。患者诊断按WHO 2016诊断标准[8]和国际骨髓纤维化研究和治疗工作组（International Working Group for Myelofibrosis Research and Treatment, IWG-MRT）诊断标准[9]。主要结局指标为总生存（Overall survival, OS）时间，定义为自芦可替尼治疗6个月后至随访截止或死亡的时间。

二、统计学处理

应用SPSS 26.0和R 4.4.2软件进行统计学分析。分类变量以例数（构成比）描述，连续变量以中位数（四分位间距）描述。采用Kaplan-Meier法绘制生存曲线，并通过Log-rank检验比较组间差异。采用RR6预后模型[4]对验证队列患者进行预后分组，并通过Loess平滑法绘制校准曲线以评估拟合优度。为比较整合分子标志物后模型的性能提升，计算了整合HMR^mt^和（或）RASp^mt^的新模型与原始RR6模型的一致性指数（Concordance index, C-index）、时间依赖性受试者工作特征曲线下面积（Area under the curve, AUC）、净重新分类改善指数（Net reclassification improvement, NRI），并绘制临床决策曲线（Decision curve analysis, DCA）。采用DeLong检验比较模型间AUC的差异。双侧*P*<0.05为差异有统计学意义。

## 结果

1. 研究对象：本中心验证队列153例MF患者中，女73例，男80例，中位年龄58（28～85）岁。PMF 114例（74.5％），post-PV MF 17例（11.1％），post-ET MF 22例（14.4％）。RR6预后模型训练队列，即意大利RUXOREL-MF研究队列，与本中心验证队列开始使用芦可替尼时的临床及实验室特征比较见表1。与训练队列相比，本中心验证队列在多项基线特征上存在显著差异。针对可进行假设检验的变量来看，本队列患者中PMF比例（74.5％对45.9％，*P*<0.001）、MF分级为3级比例（49.0％对40.4％，*P*＝0.008）更高，同时伴有体质性症状的比例（59.5％对69.9％，*P*＝0.044）、用药前3个月内有输血史比例（11.1％对23.9％，*P*＝0.002）更低。在治疗策略上，起始即使用40 mg/d剂量的患者比例较低（18.3％对37.3％，*P*<0.001）。从连续变量的分布趋势来看，本验证队列患者更年轻（58岁对67岁），中位PLT更高（249×10⁹/L对220×10⁹/L），而中位脾脏肋缘下长度相对较小（10.7 cm对12.0 cm）。

**表1:** RR6预后模型训练队列与本文验证队列开始使用芦可替尼时的临床及实验室特征

临床及实验室指标	RR6预后模型训练队列（209例）	本研究验证队列（153例）	*P*值
年龄［岁，*M*（范围）］	67（37～85）	58（28～85）	–
性别（例，男/女）	131/78	80/73	0.052
疾病类型［例（％）］			<0.001
PMF	96（45.9）	114（74.5）	
post-PV MF	78（37.3）	17（11.1）	
post-ET MF	35（16.8）	22（14.4）	
WBC［×10^9^/L，*M*（*IQR*）］	11.1（6.5～18.7）	9.9（6.1～15.1）	–
HGB［g/L，*M*（*IQR*）］	106（94～123）	108（87～123）	–
PLT［×10^9^/L，*M*（*IQR*）］	220（153～348）	249（164～355）	–
外周血原始细胞比例［％，*M*（*IQR*）］	1（0～2）	0（0～1）	–
有体质性症状［例（％）］	146（69.9）	91（59.5）	0.044
纤维化分级［例（％）］^a^			0.008
0级	1（0.5）	0（0.0）	
1级	22（12.0）	5（3.3）	
2级	86（47.0）	73（47.7）	
3级	74（40.4）	75（49.0）	
驱动基因突变类型［例（％）］^b^			0.241
JAK2	151（72.2）	105（68.6）	
CALR	31（14.8）	37（24.2）	
MPL	11（5.3）	9（5.9）	
三阴	4（1.9）	2（1.3）	
用药前3个月内有输血史［例（％）］^c^	49（23.9）	17（11.1）	0.002
脾脏肋缘下长度［cm, *M*（*IQR*）］	12.0（8.0～15.0）	10.7（8.0～14.4）	–
芦可替尼起始剂量［例（％）］			<0.001
≤15 mg/d	31（14.8）	20（13.1）	
20 mg/d	45（21.5）	47（30.7）	
30 mg/d	55（26.3）	58（37.9）	
40 mg/d	78（37.3）	28（18.3）	

**注** RR6：芦可替尼治疗6个月后反应；PMF：原发性骨髓纤维化；post-PV MF：真性红细胞增多症后骨髓纤维化；post-ET MF：原发性血小板增多症后骨髓纤维化；–：未进行假设检验。^a^训练队列183例可评估纤维化分级；^b^训练队列197例可评估驱动基因突变类型；^c^训练队列205例可评估用药前3个月内输血情况

153例MF患者中102例（66.7％）进行了NGS。共有46例（45.1％）患者检出至少1个HMR^mt^，其中15例（14.7％）患者检出≥2个HMR^mt^，HMR^mt^检出率最高的为ASXL1（42例，41.2％）；共有9例（8.8％）患者检出至少1个RASp^mt^，其中1例（1.0％）患者检出2个RASp^mt^，KRAS（3例，2.9％）、NRAS（3例，2.9％）、CBL（3例，2.9％）检出率并列最高。

2. 总体生存情况：本中心验证队列153例患者中位随访44.6（*IQR*：27.3～64.5）个月，截至末次随访，106例（69.3％）存活，94例（61.4％）仍在接受芦可替尼治疗。所有患者3年预期OS率为73.0％（95％ *CI*：65.0％～81.0％）。共有47例（30.7％）死亡，死因包括：疾病相关并发症（28例，如消化道出血、感染、血栓、心衰等）、急性白血病转化（7例）及第二原发恶性肿瘤（1例）；另有9例死因不详、2例死于意外（跌倒）；共59例（38.6％）患者停用芦可替尼，其中47例因死亡而停药；在其余12例停药患者中，6例因疗效不佳或耐药停药，5例因接受异基因造血干细胞移植停药，1例因个人原因停药。

作为对比，RR6预后模型训练队列的中位随访时间较短，为30.5（*IQR*：9.5～50.0）个月，截至末次随访，138例（66.0％）存活，中位OS时间为59.4（95％ *CI*：50.5～83.2）个月。其死因构成与本队列存在差异，共有71例（34.0％）死亡，主要为急性白血病转化（21例）、MF进展（17例）和感染（16例）。

3. RR6预后模型预测能力：采用RR6预后模型对153例MF患者进行评估，其中低危组28例（18.3％）、中危组92例（60.1％）和高危组33例（21.6％），低危组和中危组的中位OS时间皆未达到，高危组的中位OS时间为32.0（95％ *CI*：24.0～39.5）个月，差异有统计学意义（*P*＝0.012）（图2）。验证队列校准度评估见图3，第1、2、3、4、5年的校准曲线斜率分别为0.934、0.876、1.067、0.584、0.701，截距分别为0.017、0.081、0.041、0.237、0.271。提示模型在第1、2、3年的校准度良好，而在第4、5年校准度有所下降。

**图2:**
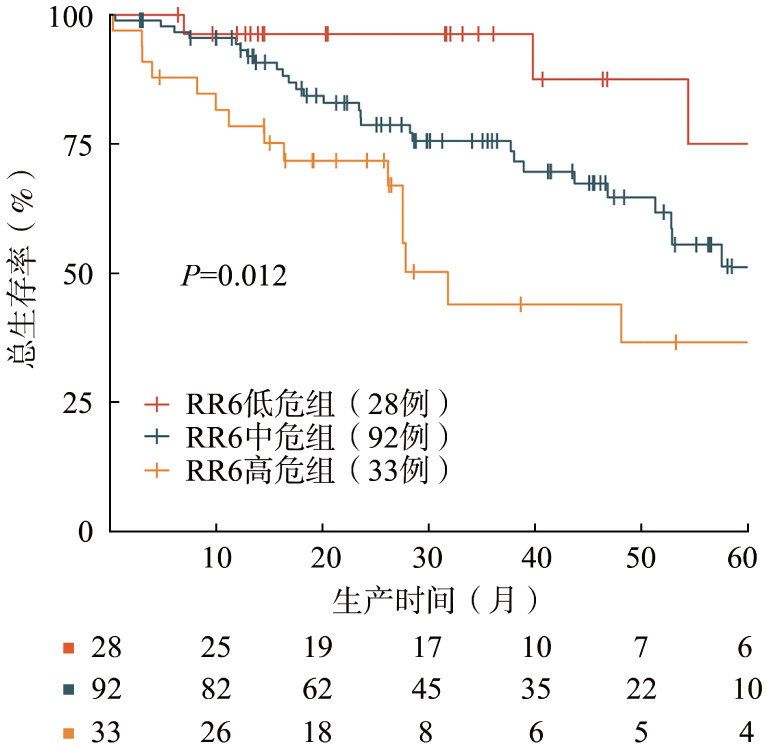
本中心验证队列153例骨髓纤维化患者根据RR6预后分组的总生存曲线 **注** RR6：芦可替尼治疗6个月后反应

**图3:**
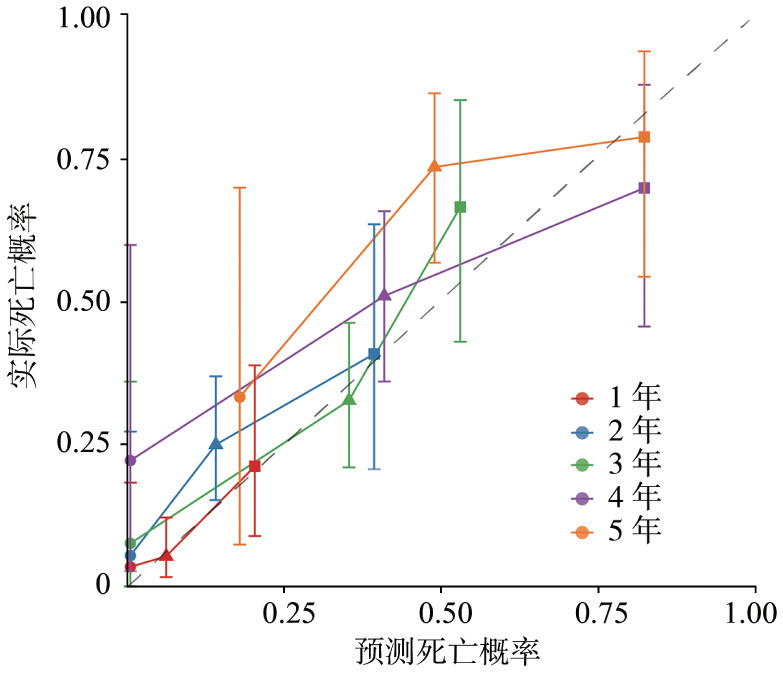
本中心验证队列153例骨髓纤维化患者根据RR6预后分组（低危组、中危组、高危组）的校准曲线 **注** RR6：芦可替尼治疗6个月后反应

4. RR6预后模型与整合模型的性能比较：在102例具有NGS数据的MF患者中，我们评估了HMR^mt^和RASp^mt^对RR6模型预测性能的增量价值，同时整合HMR^mt^与RASp^mt^的RR6模型（RR6+HMR^mt^+RASp^mt^模型）展现出最佳的综合预测性能。在模型区分度方面，该整合模型的整体C-index最高（0.735），且其时间依赖性AUC在1至5年均优于原始RR6模型及其他整合模型（表2）。尽管DeLong检验显示这些AUC的改善在统计学上不显著（各时间点均*P*>0.1），但NRI分析表明，RR6+HMR^mt^+RASp^mt^模型在预测第1年和第2年OS时，能将患者正确重新分类至更准确的风险层级，分别为0.003（95％*CI*：0.001～0.004）和0.005（95％*CI*：0.002～0.008）（*P*＝0.016、*P*<0.001）。在临床实用性方面，DCA进一步证实，应用RR6+HMR^mt^+RASp^mt^模型进行临床决策能带来更显著的净获益（图4）。

**表2:** RR6及其整合相关因素后在验证队列102例骨髓纤维化患者中的模型区分度比较

模型	C-index	AUC（95％ *CI*）				
		1年	2年	3年	4年	5年
RR6	0.649	0.72（0.56～0.88）	0.65（0.54～0.76）	0.72（0.62～0.81）	0.61（0.60～0.83）	0.59（0.57～0.87）
RR6+ HMR^mt^	0.733	0.81（0.68～1.00）	0.72（0.57～0.88）	0.77（0.64～0.91）	0.69（0.62～0.93）	0.65（0.58～0.97）
RR6+ RASp^mt^	0.735	0.81（0.63～1.00）	0.72（0.60～0.87）	0.78（0.67～0.90）	0.70（0.65～0.93）	0.66（0.61～0.97）
RR6+HMR^mt^+RASp^mt^	0.735	0.81（0.63～1.00）	0.72（0.60～0.87）	0.78（0.67～0.90）	0.71（0.65～0.92）	0.67（0.60～0.97）
HMR^mt^	0.525	0.60（0.42～0.76）	0.53（0.38～0.67）	0.47（0.33～0.61）	0.43（0.33～0.61）	0.44（0.31～0.63）
RASp^mt^	0.500	0.54（0.40～0.67）	0.49（0.41～0.56）	0.51（0.42～0.60）	0.51（0.43～0.60）	0.45（0.40～0.63）

**注** RR6：芦可替尼治疗6个月后反应；HMR^mt^：ASXL1、EZH2、IDH1、IDH2、SRSF2、U2AF1基因突变（等位基因突变频率≥2％）；RASp^mt^：NRAS、KRAS、CBL基因突变（等位基因突变频率≥2％）；C-index：一致性指数；AUC：曲线下面积

**图4:**
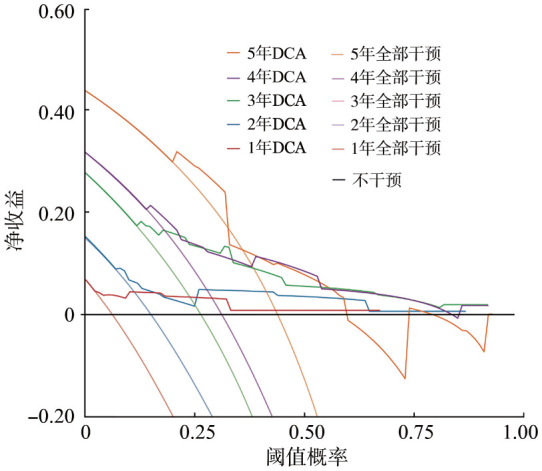
102例骨髓纤维化患者中RR6+HMR^mt^ +RASp^mt^模型的临床决策曲线 **注** RR6：芦可替尼治疗6个月后反应；HMR^mt^：ASXL1、EZH2、IDH1、IDH2、SRSF2、U2AF1基因突变（等位基因突变频率≥2％）；RASp^mt^：NRAS、KRAS、CBL基因突变（等位基因突变频率≥2％）

## 讨论

Ph^-^ MPN相关MF是起源于多能造血干细胞克隆性异常的慢性髓系肿瘤，包括PMF、post PV-MF和post ET-MF，约90％的MF患者存在JAK2、CALR和MPL驱动基因突变[10]–[13]，骨髓病理以MF为特征，临床表现以贫血、体质性症状、髓外造血、进行性肝脾肿大、进展为急性白血病、OS期缩短等为特征[14]。JAK抑制剂可通过减少JAK2和STAT磷酸化从而减少细胞增殖并诱导细胞凋亡[15]。2011年，JAK1/2抑制剂芦可替尼获美国食品与药品监督管理局（FDA）批准，用于治疗国际预后积分系统（IPSS）较高危组MF患者。2017年，该药物经中国国家药品监督管理局批准在国内上市，标志着我国MPN相关MF正式进入芦可替尼治疗时代。多数患者使用芦可替尼6个月内，脾脏肿大以及体质性症状有不同程度改善[16]。且既往研究表明，无论是在临床试验还是在真实世界中，芦可替尼与对照组（安慰剂或最佳可用疗法）相比可改善MPN相关MF患者的OS[17]–[20]。部分MF患者在芦可替尼治疗早期可能因血细胞减少等而不耐受，需减低剂量，从而影响疗效，或早期即出现对芦可替尼原发耐药；即使是一开始对芦可替尼反应良好的患者，也可能会逐渐丧失疗效而导致停药。既往已有文献报道芦可替尼停药后的MF患者中位OS时间为4～32个月[21]–[24]。如何能更早地识别出这部分患者，及时调整治疗方案以提高疗效和延长生存至关重要。

虽然2010年IWG-MRT根据PMF患者的随访数据提出了动态国际预后积分系统（Dynamic International Prognostic Scoring System, DIPSS），但DIPSS[25]因提出时间较早，构建模型时未纳入接受芦可替尼治疗的PMF患者。继发于PV和ET的MF预后模型（Myelofibrosis Secondary to PV and ET-Prognostic Model, MYSEC-PM）[26]纳入的所有post-PV/ET MF患者的诊断时间为1981–2015年，虽未对治疗方案进行详细说明，但其中75.6％的患者接受了减细胞治疗，提示这些患者很可能接受了包括芦可替尼在内的不同的治疗方案。Maffioli等[4]对209例接受芦可替尼治疗6个月后的MF患者使用进行预后分组，因芦可替尼治疗的影响，DIPSS和MYSEC-PM预测能力下降，提示上述预后积分系统可能并不适用于接受芦可替尼治疗的MF患者。因此，Maffioli等[4]建立了RR6预后模型；根据该模型进行预后分组，低危、中危和高危组患者的中位OS时间分别为未达到、61（95％*CI*：43～80）个月和33（95％*CI*：21～50）个月（*P*<0.001）。本研究中我们采用RR6预后模型对我国153例接受芦可替尼治疗的MF患者进行预后分组，通过外部验证证实RR6具有准确的预后预测能力（*P*＝0.012），校准曲线提示RR6模型在第1、2、3年的校准度良好。上述研究结果与既往国外相关研究结果一致[5],[27]–[28]，表明RR6模型亦可用于我国接受芦可替尼治疗的MF患者的生存预后评估。在临床上，对于应用芦可替尼治疗的MF患者，可以在芦可替尼治疗6个月后即对患者应用RR6模型进行评估，尽早识别出芦可替尼治疗的高危组患者，及时调整治疗方案或尽早进行异基因造血干细胞移植。

除驱动基因突变外，既往研究发现ASXL1、EZH2、IDH1、IDH2、SRSF2和U2AF1等非驱动基因突变与MPN相关MF患者不良预后有关[10],[29]，上述突变被称为HMR^mt^。RAS信号通路相关基因包括RAS基因（KRAS、NRAS和HRAS）、信号级联放大中RAS上游的基因（CBL、FLT3等），以及参与RAS信号通路调控的基因（PTPN11、NF1等）[30]。Coltro等[31]发现NRAS、KRAS和CBL基因突变不仅与MF患者不良预后相关，并且可能预示着MF患者对JAK抑制剂治疗的反应差，并在后续研究中将其称为RASp^mt^［5]。Maffioli等[4]考虑到NGS在临床实践中尚未普及，因此在构建RR6模型时并未纳入基因突变特征。Coltro等[5]不仅在RR6模型上整合了HMR^mt^和RASp^mt^，并证实其对生存预后的预测能力得到提高，RR6+HMR^mt^+RASp^mt^模型对生存预后的预测能力表现最优[5]。本研究中，我们计算出整合HMR^mt^和（或）RASp^mt^后的RR6的C-index和时间依赖性AUC都高于原始RR6模型，但DeLong检验显示AUC的差异均无统计学意义（均*P*>0.1）。NRI显示RR6+HMR^mt^+RASp^mt^模型与RR6模型相比，在1、2年的预测价值有所提高（*P*＝0.016、*P*<0.001）；DCA亦显示RR6+HMR^mt^+RASp^mt^模型具有明显的临床净获益。上述结果提示整合HMR^mt^和RASp^mt^后的RR6预后模型或可进一步提高对接受芦可替尼治疗的MF患者生存预后的预测能力。

综上所述，本研究在我国接受芦可替尼治疗的MPN相关MF病例中验证了RR6模型的生存预后评估价值。经6个月芦可替尼治疗后可使用RR6模型对患者进行生存预后分组，对于中危组及高危组患者，应采取更积极的治疗策略，如更换其他JAK抑制剂、异基因造血干细胞移植或参加临床试验。随着NGS的普及，相继有研究结果证实分子遗传学信息在指导MF患者治疗方案的选择和预后评估等方面的重要性。我们亦证实RR6整合HMR^mt^和RASp^mt^后的预后预测能力或可进一步提升，为应用芦可替尼治疗的MPN相关MF患者个体化治疗和动态预后监测提供可能。本研究存在若干局限性，具体如下：①为单中心回顾性研究，不可避免地存在选择偏倚；②样本量有限，容易导致模型过拟合；③随访时间较短、总事件数少，导致模型区分度与校准度的估计值置信区间较宽，影响了评估结果的精确性；④对于重要变量的缺失数据，我们采取了完整案例分析法，但该方法只适合随机缺失数据，不适用于非随机缺失数据。未来需通过多中心前瞻性研究，在更大样本中进一步验证。
